# New estimations on the upper bounds for the nuclear norm of a tensor

**DOI:** 10.1186/s13660-018-1861-1

**Published:** 2018-10-11

**Authors:** Xu Kong, Jicheng Li, Xiaolong Wang

**Affiliations:** 1 School of Mathematical Sciences, Liaocheng University, Liaocheng, China; 2 School of Mathematics and Statistics, Xi’an Jiaotong University, Xi’an, China; 3 School of Science, Northwestern Polytechnical University, Xi’an, China

**Keywords:** 15A18, 15A69, Tensor, Nuclear norm, Orthogonal rank, Upper bound

## Abstract

Using the orthogonal rank of the tensor, a new estimation method for the upper bounds on the nuclear norms is presented and some new tight upper bounds on the nuclear norms are established. Taking into account the structure information of the tensor, an important factor affecting the upper bounds is discussed and some corresponding properties related to the nuclear norms are given. Meanwhile, some new results of the upper bounds on the nuclear norms are obtained.

## Introduction

A tensor is a multidimensional array that can provide a natural and convenient way for representing the multidimensional data such as discrete forms of multivariate functions, images, video sequences, and so on [1, 2, 10, 14]. With the successful and widespread use of the matrix nuclear norm (a sum of the singular values) in the information recovery, the research on the nuclear norm of the tensor (see Definition 2.3 in Sect. 2) has been a hot topic in both the theory and applications [5–8].

A natural problem is how to compute the nuclear norm of a tensor. Unfortunately, compared with the matrix nuclear norm, the nuclear norm of a tensor is closely related to the number field, and the computation of the tensor nuclear norm is NP-hard [5]. Thus, exploring some simple polynomial-time computable upper bounds on the nuclear norm is very important.

Relating to the nuclear norm of a tensor, Friedland and Lim established the following upper bound through the Frobenius norm of this tensor in [5].

### Theorem 1.1

([5])

*Let* $\mathcal{X}\in \mathbb{R}^{n_{1}\times \cdots \times n_{D}}$. *Then*

$$\begin{aligned} \Vert \mathcal{X} \Vert _{\ast }\leq \sqrt{ \prod_{i=1}^{D}n_{i}} \Vert \mathcal{X} \Vert _{F}. \end{aligned}$$

In [8], Hu established a tighter upper bound.

### Theorem 1.2

(Lemma 5.1 in [8])

*Let* $\mathcal{X}\in \mathbb{R}^{n_{1} \times \cdots \times n_{D}}$. *Then*

1 $$\begin{aligned} \Vert \mathcal{X} \Vert _{\ast }\leq \sqrt{ \frac{\prod_{i=1}^{D}n_{i}}{ \max \{n_{1},\ldots, n_{D}\}}} \Vert \mathcal{X} \Vert _{F}. \end{aligned}$$

Furthermore, Hu established another upper bound on the nuclear norm of a tensor through the nuclear norms of its unfolding matrices.

### Theorem 1.3

(Theorem 5.2 in [8])

*Let* $\mathcal{X}\in \mathbb{R}^{n_{1} \times \cdots \times n_{D}}$. *Then*

2 $$\begin{aligned} \Vert \mathcal{X} \Vert _{\ast }\leq \sqrt{ \frac{\prod_{i=2}^{D}n_{i}}{ \max \{n_{2},\ldots, n_{D}\}}} \Vert \mathbf{X}_{(1)} \Vert _{\ast }. \end{aligned}$$

In this paper, we present some new upper bounds on the nuclear norms through using the orthogonal rank of the tensor [9, 12]. Furthermore, taking into account the structure information of the tensor, some new results of the upper bounds on the nuclear norms are obtained.

Since the spectral norm and nuclear norm of a tensor are closely related to the number field [6], for the sake of simplicity, we always assume that the tensors discussed are nonzero real tensors, and unless mentioned otherwise, we just discuss the spectral and the nuclear norm over the real field. Some corresponding notations are as follows: a tensor is denoted by the calligraphic letter (e.g., $\mathcal{X}$), while the scalar is denoted by the plain letter, and matrices and vectors are denoted by bold letters (e.g., **X** and **x**).

The rest of the paper is organized as follows. In Sect. 2, we recall some definitions and related results which are needed for the subsequent sections. In Sect. 3, we present the upper bounds on the nuclear norms of general tensors and discuss the factor affecting the upper bounds. Finally, some conclusions are made in Sect. 4.

## Notations and preliminaries

This section is devoted to reviewing some conceptions and results related to tensors, which are needed for the following sections.

Firstly, we discuss the unfolding matrix or matrix representation of a tensor.

Let $\mathcal{X}=(x_{i_{1}\cdots i_{D}})\in \mathbb{R}^{n_{1}\times \cdots \times n_{D}}$. Then, by organizing several indexes of $\mathcal{X}$ and the remaining indexes of $\mathcal{X}$ as the row index and the column index, respectively, the tensor $\mathcal{X}$ can be reshaped into a matrix form [13]. Especially, if the number of row index is equal to one, then we get the mode-*d* matricization $\mathbf{X}_{(d)}$, the columns of which are the mode-*d* fibers of the tensor (obtained by fixing every coordinate except one, $[x_{i_{1}\cdots i_{d-1}1i_{d+1}\cdots i_{D}},\ldots, x_{i_{1}\cdots i_{d-1}n_{d}i_{d+1}\cdots i_{D}}]^{T}$), arranged in a cyclic ordering; see [3] for details. Contrary to the operation above, a matrix can also be reshaped into a tensor by using the opposite operation.

In what follows, we review some definitions of the tensor norms.

### Definition 2.1

([3])

Let $\mathcal{X}=(x_{i_{1}\cdots i_{D}}) \in \mathbb{R}^{n_{1}\times \cdots \times n_{D}}$, the Frobenius norm or Hilbert–Schmidt norm of the tensor $\mathcal{X}$ is defined as

$$\begin{aligned} \Vert \mathcal{X} \Vert _{F}=\sqrt{\langle \mathcal{X}, \mathcal{X}\rangle }= \Biggl( \sum_{i_{1}=1}^{n_{1}} \cdots \sum_{i_{D}=1}^{n_{D}}x_{i_{1} \cdots i_{D}}^{2} \Biggr) ^{1/2}. \end{aligned}$$

### Definition 2.2

([6])

Let “∘” denote the outer product operation. The spectral norm of $\mathcal{X}\in \mathbb{R}^{n_{1}\times \cdots \times n_{D}}$ is defined as

$$\begin{aligned} \Vert \mathcal{X} \Vert _{2}=\max \bigl\{ \bigl\langle \mathcal{X}, \mathbf{x}^{(1)} \circ \cdots \circ \mathbf{x}^{(D)}\bigr\rangle : \mathbf{x}^{(d)}\in \mathbb{R}^{n_{d}}, \bigl\Vert \mathbf{x}^{(d)} \bigr\Vert _{2}=1, 1\leq d\leq D\bigr\} . \end{aligned}$$

Furthermore, $\Vert \mathcal{X}\Vert _{2}$ is equal to the Frobenius norm of the best rank-one approximation of the tensor $\mathcal{X}$.

Similar to the matrix case, the nuclear norm can be defined through the dual norm of the spectral norm.

### Definition 2.3

([6])

Let $\mathcal{X}\in \mathbb{R}^{n_{1}\times \cdots \times n_{D}}$, the nuclear norm of $\mathcal{X}$ is defined as the dual norm of the spectral norm. That is,

3 $$\begin{aligned} \Vert \mathcal{X} \Vert _{\ast }:=\max \bigl\{ \langle \mathcal{X},\mathcal{Y}\rangle :\mathcal{Y}\in \mathbb{R}^{n_{1}\times \cdots \times n_{D}}, \Vert \mathcal{Y} \Vert _{2}=1\bigr\} . \end{aligned}$$

For the nuclear norm defined by (3), it can be shown that

$$\begin{aligned} \Vert \mathcal{X} \Vert _{\ast } =&\min \Biggl\{ \sum _{p=1}^{P}{ \vert \lambda_{p} \vert }: \mathcal{X}=\sum_{p=1}^{P} \lambda_{p}\mathbf{x}_{p}^{(1)} \circ \cdots \circ \mathbf{x}_{p}^{(D)}, \\ & \bigl\Vert \mathbf{x}_{p}^{(d)} \bigr\Vert _{2}=1, \mathbf{x}_{p}^{(d)}\in \mathbb{R}^{n_{d}}, \lambda_{p} \in \mathbb{R}, P\in \mathbb{N} \Biggr\} . \end{aligned}$$

Another important concept related to the matrix and the tensor is the mode-*d* multiplication.

### Definition 2.4

([3])

Let $\mathcal{ X}=(x_{i_{1}\cdots i_{D}}) \in \mathbb{R}^{n_{1}\times \cdots \times n_{D}}$. Then the mode-*d* multiplication of $\mathcal{X}$ by the matrix $\mathbf{U}=(u_{ij})\in \mathbb{R}^{n'_{d}\times n_{d}}$ is defined by

$$\begin{aligned} (\mathcal{X}\times_{d}\mathbf{U})_{i_{1}\cdots i_{d-1}i'_{d}i_{d+1} \cdots i_{D}} =\sum _{i_{d}=1}^{n_{d}}x_{i_{1}\cdots i_{d-1}i_{d}i_{d+1} \cdots i_{D}}u_{i'_{d}i_{d}}, \quad 1\leq d \leq D. \end{aligned}$$

It should be mentioned that the mode-*d* multiplication is also available for $n_{d}^{\prime}=1$.

Furthermore, let

$$\begin{aligned} \bigl(\mathbf{W}^{(1)},\ldots, \mathbf{W}^{(D)}\bigr)\cdot \mathcal{X}=\mathcal{X} \times_{1}{\mathbf{W}}^{(1)}\times \cdots \times_{D}{\mathbf{W}}^{(D)}. \end{aligned}$$

If for all $1\leq d\leq D$ the matrices $\mathbf{W}^{(d)}$ are orthogonal matrices ($\mathbf{W}^{(d)}{\mathbf{W}}^{(d)^{T}}$ is an identity matrix), then $(\mathbf{W}^{(1)},\ldots, \mathbf{W}^{(D)})\cdot \mathcal{X}$ is called a multi-linear orthogonal transformation of the tensor $\mathcal{X}$.

Finally in this section, we introduce the tool used in the paper for the estimation of the upper bounds.

### Definition 2.5

([9])

The orthogonal rank of $\mathcal{X}\in \mathbb{R} ^{n_{1}\times \cdots n_{D}} $ is defined as the smallest number *R* such that

4 $$\begin{aligned} \mathcal{X}=\sum_{r=1}^{R} \mathcal{U}_{r}, \end{aligned}$$

where $\mathcal{U}_{r}$ ($1\leq r\leq R$) are rank-one tensors such that $\langle \mathcal{U}_{r_{1}},\mathcal{U}_{r_{2}}\rangle =0$, ($r_{1}\neq r_{2}$) for $1\leq r_{1}\leq R$ and $1\leq r_{2}\leq R$.

The decomposition of $\mathcal{X}$ given by (4) is also called the orthogonal decomposition of the tensor $\mathcal{X}$.

For the orthogonal rank of a tensor, the following conclusion is true.

### Theorem 2.1

([11])

*Let* $n_{1}\leq \cdots \leq n_{D}$. *Then*, *for any* $\mathcal{X}\in \mathbb{R}^{n_{1}\times \cdots \times n_{D}}$, *it holds*

$$\begin{aligned} r_{\perp }(\mathcal{X}) \leq \prod_{i=1}^{D-1}n_{i}, \end{aligned}$$

*where* $r_{\perp }(\mathcal{X})$ *denotes the orthogonal rank of* $\mathcal{X}$.

Noting the fact that indices relabeling does not change the tensor orthogonal rank, then by Theorem 2.1, we have that, for any $\mathcal{X}\in \mathbb{R}^{n_{1}\times \cdots \times n _{D}}$, it holds

5 $$\begin{aligned} r_{\perp }(\mathcal{X}) \leq \frac{\prod_{i=1}^{D}n_{i}}{\max \{n_{1},\ldots, n_{D}\}}. \end{aligned}$$

Especially, for the third order tensor, the following result was established in [11].

### Lemma 2.1

([11])

*Let* $n\geq 2$. *Then*, *for any* $\mathcal{X}\in \mathbb{R}^{n\times n \times 2}$, *the following holds*:

$$\begin{aligned} r_{\perp }(\mathcal{X}) \leq \textstyle\begin{cases} 2n-1,& \textit{if } n \textit{ is odd}; \\ 2n, & \textit{if } n \textit{ is even}. \end{cases}\displaystyle \end{aligned}$$

## Upper bounds of the nuclear norm

In this section, we discuss the upper bounds on the nuclear norm of a tensor. Meanwhile, some properties and polynomial-time computable bounds related to the nuclear norm will be given.

### Upper bounds given by the Frobenius norm

In this subsection, we use the orthogonal rank of a general tensor to establish the upper bounds on the nuclear norm through the Frobenius norm of this tensor.

#### Theorem 3.1

*Let* $\mathcal{X}\in \mathbb{R}^{n_{1}\times \cdots \times n_{D}}$. *Suppose that*

$$\begin{aligned} R=\max_{\mathcal{Y}\in \mathbb{R}^{n_{1}\times \cdots \times n_{D}}} \bigl\{ \operatorname{rank}_{\bot }(\mathcal{Y})\bigr\} . \end{aligned}$$

*Then*

6 $$\begin{aligned} \Vert \mathcal{X} \Vert _{\ast }\leq \sqrt{R} \Vert \mathcal{X} \Vert _{F}. \end{aligned}$$

#### Proof

Let $\mathcal{Y}\in \mathbb{R}^{n_{1}\times \cdots \times n_{D}}$ be an arbitrary nonzero tensor and the orthogonal rank of $\mathcal{Y}$ be $R_{y}$. Suppose that

$$\begin{aligned} \mathcal{Y}=\sum_{r=1}^{R_{y}} \mathcal{U}_{r} \end{aligned}$$

is the orthogonal rank decomposition of the tensor $\mathcal{Y}$, where $R_{y}\leq R$.

Then, according to the properties of the orthogonal rank decomposition and the best rank-one approximation, we have

7 $$\begin{aligned} \Vert \mathcal{Y} \Vert _{2}\geq \max _{1\leq r\leq R_{y}}\bigl\{ \Vert \mathcal{U}_{r} \Vert _{F}\bigr\} \end{aligned}$$

and

8 $$\begin{aligned} \Vert \mathcal{Y} \Vert _{F}^{2}=\sum _{r=1}^{R_{y}} \Vert \mathcal{U}_{r} \Vert _{F}^{2}. \end{aligned}$$

Without loss of generality, suppose that

$$\begin{aligned} \Vert \mathcal{U}_{1} \Vert _{F}=\max _{1\leq r\leq R_{y}}\bigl\{ \Vert \mathcal{U}_{r} \Vert _{F}\bigr\} . \end{aligned}$$

Then it follows from (7) and (8) that

9 $$\begin{aligned} \begin{aligned}[b] \biggl\langle \mathcal{X},\frac{\mathcal{Y}}{ \Vert \mathcal{Y} \Vert _{2}} \biggr\rangle & \leq \Vert \mathcal{X} \Vert _{F}\frac{ \Vert \mathcal{Y} \Vert _{F}}{ \Vert \mathcal{Y} \Vert _{2}} \\ &\leq \Vert \mathcal{X} \Vert _{F}\frac{\sqrt{\sum_{r=1}^{R_{y}} \Vert \mathcal{U}_{r} \Vert _{F}^{2}}}{ \Vert \mathcal{U}_{1} \Vert _{F}} \\ &\leq \Vert \mathcal{X} \Vert _{F}\frac{\sqrt{R_{y} \Vert \mathcal{U}_{1} \Vert _{F} ^{2}}}{ \Vert \mathcal{U}_{1} \Vert _{F}} \\ &=\sqrt{R_{y}} \Vert \mathcal{X} \Vert _{F} \\ &\leq \sqrt{R} \Vert \mathcal{X} \Vert _{F}. \end{aligned} \end{aligned}$$

Thus, according to the arbitrariness of $\mathcal{Y}$ and (9), we get

$$\begin{aligned} \max_{\mathcal{Y}\in \mathbb{R}^{n_{1}\times \cdots \times n_{D}}} \biggl\{ \biggl\langle \mathcal{X}, \frac{\mathcal{Y}}{ \Vert \mathcal{Y} \Vert _{2}} \biggr\rangle \biggr\} \leq \sqrt{R} \Vert \mathcal{X} \Vert _{F}. \end{aligned}$$

Noting the definition of the nuclear norm (Definition 2.3), the conclusion is established. □

#### Remark 3.1

Comparing the upper bound given by (6) with the upper bound given by (1), which is obtained in [8], the new upper bound given by (6) is tighter.

Actually, it follows from (5) that the upper bound given by (6) improves the upper bound given by (1).

More specifically, we present a simple example to show that the upper bound given by Theorem 3.1 not only can be tighter than the upper bound given by (1) but also a sharp bound.

#### Example 3.1

Let

$$A=\left[\begin{array}{ccc} 0 & 1 & 0\\ 1 & 0 & 0\\ 0 & 0 & 0 \end{array}\;|\;\begin{array}{ccc} -1 & 0 & 0\\ 0 & 1 & 0\\ 0 & 0 & 1 \end{array}\right]\in R^{3\times 3\times 2}.$$

By Theorem 3.1 and Lemma 2.1, we get

$$\begin{aligned} \Vert \mathcal{A} \Vert _{\ast }\leq \sqrt{5} \sqrt{1^{2}+1^{2}+(-1)^{2}+1^{2}+1^{2}}=5< \sqrt{\frac{3\times 3 \times 2}{3}}\sqrt{5}=\sqrt{30}. \end{aligned}$$

This means that the upper bound given by (3.1) is tighter than the upper bound given by (1).

Furthermore, by a simple computation, we get $\Vert \mathcal{A}\Vert _{2}=1$. Then it follows from the definition of the nuclear norm that

$$\begin{aligned} \Vert \mathcal{A} \Vert _{\ast }\geq \biggl\langle \mathcal{A}, \frac{ \mathcal{A}}{ \Vert \mathcal{A} \Vert _{2}} \biggr\rangle =\frac{ \Vert \mathcal{A} \Vert _{F}^{2}}{ \Vert \mathcal{A} \Vert _{2}}=\frac{5}{1}=5. \end{aligned}$$

Thus, it holds

$$\begin{aligned} \Vert \mathcal{A} \Vert _{\ast }=5. \end{aligned}$$

Actually,

10 $$\begin{aligned} \begin{aligned}[b] \mathcal{A}&=\mathbf{e}_{1;3}\circ \mathbf{e}_{2;3}\circ \mathbf{e} _{1;2}+\mathbf{e}_{2;3} \circ \mathbf{e}_{1;3}\circ \mathbf{e}_{1;2} +(- \mathbf{e}_{1;3})\circ \mathbf{e}_{1;3}\circ \mathbf{e}_{2;2}+ \mathbf{e}_{2;3}\circ \mathbf{e}_{2;3} \circ \mathbf{e}_{2;2} \\ &\quad{} +\mathbf{e}_{3;3}\circ \mathbf{e}_{3;3}\circ \mathbf{e}_{2;2} \end{aligned} \end{aligned}$$

is a nuclear decomposition of $\mathcal{A}$, where $\mathbf{e}_{1;3}=[1, 0, 0]^{T}$, $\mathbf{e}_{2; 3}=[0, 1, 0]^{T}$, $\mathbf{e}_{3; 3}=[0, 0, 1]^{T}$, $\mathbf{e}_{1; 2}=[1, 0]^{T}$, and $\mathbf{e}_{2; 2}=[0, 1]^{T}$. Since

$$\begin{aligned} \begin{aligned} \langle \mathcal{A}, \mathbf{e}_{1;3}\circ \mathbf{e}_{2;3}\circ \mathbf{e}_{1;2}\rangle &=\langle \mathcal{A}, \mathbf{e}_{2;3}\circ \mathbf{e}_{1;3}\circ \mathbf{e}_{1;2}\rangle =\bigl\langle \mathcal{A}, (- \mathbf{e}_{1;3})\circ \mathbf{e}_{1;3}\circ \mathbf{e}_{2;2}\bigr\rangle \\ & =\langle \mathcal{A}, \mathbf{e}_{2;3} \circ \mathbf{e}_{2;3}\circ \mathbf{e}_{2;2}\rangle =\langle \mathcal{A}, \mathbf{e}_{3;3}\circ \mathbf{e}_{3;3} \circ \mathbf{e}_{2;2}\rangle , \end{aligned} \end{aligned}$$

then, according to the sufficient and necessary conditions of the nuclear norm decomposition obtained in [6], we get that (10) is a nuclear decomposition of $\mathcal{A}$.

This also means that the upper bound given by Theorem 3.1 is a sharp upper bound of the nuclear norm.

### Upper bounds given by nuclear norms of the unfolding matrices of a tensor

In this subsection, we present a new way to establish the upper bounds on the nuclear norm of a tensor through the nuclear norms of the unfolding matrices of this tensor.

#### Theorem 3.2

*Let* $\mathcal{X}\in \mathbb{R}^{n_{1}\times \cdots \times n_{D}}$. *Suppose that*

$$\begin{aligned} \tilde{R}= \max_{\mathcal{Y}\in \mathbb{R}^{n_{2}\times \cdots \times n_{D}}}\bigl\{ \operatorname{rank} _{\bot }( \mathcal{Y})\bigr\} . \end{aligned}$$

*Then*

11 $$\begin{aligned} \Vert \mathcal{X} \Vert _{\ast }\leq \sqrt{\tilde{R}} \Vert \mathbf{X}_{(1)} \Vert _{\ast }. \end{aligned}$$

#### Proof

Let the singular value decomposition of the matrix $\mathbf{X}_{(1)}$ be

12 $$\begin{aligned} \mathbf{X}_{(1)}=\sigma_{1} \mathbf{u}_{1}\mathbf{v}_{1}^{T}+\cdots + \sigma_{S}\mathbf{u}_{S}\mathbf{v}_{S}^{T}, \end{aligned}$$

where $\mathbf{u}_{s}\in \mathbb{R}^{n_{1}}$, $\mathbf{v}_{s}\in \mathbb{R}^{n_{2}\times \cdots \times n_{D}}$, $\Vert \mathbf{u}_{s}\Vert _{2}=1$, $\Vert \mathbf{v}_{s}\Vert _{2}=1$, $\sigma_{s}>0$, and $1\leq s \leq S$.

Then equality (12) can be expressed as the following form:

13 $$\begin{aligned} \mathcal{X}=\sigma_{1}\mathbf{u}_{1}\circ \mathcal{V}_{1}+\cdots + \sigma_{S}\mathbf{u}_{S} \circ \mathcal{V}_{S}, \end{aligned}$$

where $\mathcal{V}_{s}\in \mathbb{R}^{n_{2}\times \cdots \times n_{D}}$ are obtained by reordering the vector $\mathbf{v}_{s}$ into $(D-1)$th order tensor with a certain order, $1\leq s\leq S$. Suppose that the orthogonal rank decomposition of $\mathcal{V}_{s}$ is

$$\begin{aligned} \mathcal{V}_{s}=\mathbf{v}_{1}^{(1,s)}\circ \cdots \circ \mathbf{v} _{1}^{(D-1, s)}+\cdots +\mathbf{v}_{P_{s}}^{(1, s)} \circ \cdots \circ \mathbf{v}_{P_{s}}^{(D-1, s)}, \quad 1\leq s\leq S. \end{aligned}$$

Then, by taking the expression of $\mathcal{V}_{s}$ into the right-hand side of (13), we get

14 $$\begin{aligned} \begin{aligned}[b] \mathcal{X}&=\sigma_{1}\sum _{i=1}^{P_{1}} \mathbf{u}_{1}\circ \mathbf{v}_{i}^{(1,1)}\circ \cdots \circ \mathbf{v}_{i}^{(D-1, 1)}+ \cdots + \sigma_{S}\sum_{i=1}^{P_{S}} \mathbf{u}_{1}\circ \mathbf{v} _{i}^{(1,S)}\circ \cdots \circ \mathbf{v}_{i}^{(D-1, S)} \\ &=\sigma_{1}\sum_{i=1}^{P_{1}} \bigl( \bigl\Vert \mathbf{v}_{i}^{(1,1)} \bigr\Vert _{2} \cdots \bigl\Vert \mathbf{v}_{i}^{(D-1, 1)} \bigr\Vert _{2} \bigr) \mathbf{u}_{1}\circ \frac{ \mathbf{v}_{i}^{(1,1)}}{ \Vert \mathbf{v}_{i}^{(1,1)} \Vert _{2}} \circ \cdots \circ \frac{\mathbf{v}_{i}^{(D-1, 1)}}{ \Vert \mathbf{v}_{i}^{(D-1, 1)} \Vert _{2}}+\cdots \\ & \quad{} + \sigma_{S}\sum_{i=1}^{P_{S}} \bigl( \bigl\Vert \mathbf{v}_{i}^{(1,s)} \bigr\Vert _{2} \cdots \bigl\Vert \mathbf{v}_{i}^{(D-1, s)} \bigr\Vert _{2} \bigr) \mathbf{u}_{1}\circ \frac{ \mathbf{v}_{i}^{(1,S)}}{ \Vert \mathbf{v}_{i}^{(1,S)} \Vert _{2}} \circ \cdots \circ \frac{\mathbf{v}_{i}^{(D-1, S)}}{ \Vert \mathbf{v}_{i}^{(D-1, S)} \Vert _{2}}. \end{aligned} \end{aligned}$$

Noting that

15 $$\begin{aligned} \Vert \mathcal{V}_{s} \Vert _{F}^{2}= \sum_{i=1}^{P_{s}} \bigl\Vert \mathbf{v}_{i}^{(1,s)} \circ \cdots \circ \mathbf{v}_{i}^{(D-1, s)} \bigr\Vert _{F}^{2}=\sum_{i=1}^{P _{s}} \bigl\Vert \mathbf{v}_{i}^{(1,s)} \bigr\Vert _{F}^{2}\cdots \bigl\Vert \mathbf{v}_{i}^{(D-1, s)} \bigr\Vert _{F}^{2}= \Vert \mathbf{v}_{s} \Vert _{F}^{2}=1, \end{aligned}$$

where $P_{s}\leq \tilde{R}$, $1\leq s\leq S$, then it follows from the definition of the nuclear norm and (14) that

$$\begin{aligned} \begin{aligned} \Vert \mathcal{X} \Vert _{\ast }&\leq \sigma_{1}\sum_{i=1}^{P_{1}} \bigl\Vert \mathbf{v} _{i}^{(1,1)} \bigr\Vert _{2} \cdots \bigl\Vert \mathbf{v}_{i}^{(D-1, 1)} \bigr\Vert _{2}+\cdots + \sigma_{S}\sum_{i=1}^{P_{S}} \bigl\Vert \mathbf{v}_{i}^{(1,s)} \bigr\Vert _{2} \cdots \bigl\Vert \mathbf{v}_{i}^{(D-1, s)} \bigr\Vert _{2} \\ &\leq \sigma_{1}\sqrt{P_{1}}+\cdots +\sigma_{S} \sqrt{P_{S}}\quad \bigl(\text{by (15) and Cauchy-Schwarz inequality}\bigr) \\ &\leq \sigma_{1}\sqrt{\tilde{R}}+\cdots +\sigma_{S}\sqrt{ \tilde{R}} \\ &=\sqrt{\tilde{R}} \Vert \mathbf{X}_{(1)} \Vert _{\ast }. \end{aligned} \end{aligned}$$

 □

#### Remark 3.2

Comparing the upper bound given by (11) with the upper bound given by (2), which is obtained in [8], the new upper bound given by (11) is smaller.

Actually, it follows from inequality (5) that the upper bound given by (11) improves the upper bound given by (2).

Similar to the discussion of Hu [8], the upper bounds can also be obtained by other unfolding ways and further improved by considering the multi-linear ranks of a tensor (ranks of the unfolding matrices).

#### Corollary 3.1

*Let* $\mathcal{X}\in \mathbb{R}^{n_{1}\times n_{2}\times n_{3}}$ *and* $r_{d}=\operatorname{rank}(\mathbf{X}_{(d)})$, $1\leq d\leq 3$. *Then*

$$\begin{aligned} \Vert \mathcal{X} \Vert _{\ast }\leq \frac{\sqrt{\min \{r_{2}, r_{3}\}} \Vert {\mathbf{X}}_{(1)} \Vert _{\ast }+ \sqrt{\min \{r_{3}, r_{1}\}} \Vert {\mathbf{X}}_{(2)} \Vert _{\ast }+\sqrt{\min \{r_{1}, r_{2}\}} \Vert {\mathbf{X}}_{(3)} \Vert _{\ast }}{3}. \end{aligned}$$

#### Proof

According to the conditions of the corollary and the higher order singular value decomposition of the tensor [3], the tensor $\mathcal{X}$ can be expressed as

$$\begin{aligned} \mathcal{X}=\bigl(\mathbf{W}^{(1)}, \mathbf{W}^{(2)}, \mathbf{W}^{(3)}\bigr)\cdot \tilde{\mathcal{X}}, \end{aligned}$$

where $\tilde{\mathcal{X}}\in \mathbb{R}^{r_{1}\times r_{2}\times r _{3}}$ and $\mathbf{W}^{(d)}\in \mathbb{R}^{n_{d}\times r_{d}}$ satisfying that $\mathbf{W}^{(d)^{T}}{\mathbf{W}}^{(d)}$ is an identity matrix for all $1\leq d\leq 3$.

By the definition of the tensor nuclear norm (Definition 2.3), one can easily verify the following conclusions:

16 $$\begin{aligned} \Vert \tilde{\mathcal{X}} \Vert _{\ast }= \Vert \mathcal{X} \Vert _{\ast }, \end{aligned}$$

and

17 $$\begin{aligned} \Vert \tilde{\mathbf{X}}_{(1)} \Vert _{\ast }= \Vert {\mathbf{X}}_{(1)} \Vert _{\ast }. \end{aligned}$$

It follows from Theorem 3.2 and (17) that

$$\begin{aligned} \Vert \tilde{\mathcal{X}} \Vert _{\ast }\leq \sqrt{ \max _{\mathcal{Y}\in \mathbb{R}^{r_{2}\times r_{3}}}\bigl\{ \operatorname{rank}_{\bot }( \mathcal{Y})\bigr\} } \Vert \tilde{\mathbf{X}}_{(1)} \Vert _{\ast }\leq \sqrt{ \min \{r_{2}, r_{3}\}} \Vert {\mathbf{X}}_{(1)} \Vert _{\ast }. \end{aligned}$$

Noting (16), we get

$$\begin{aligned} \Vert \mathcal{X} \Vert _{\ast }\leq \sqrt{\min \{r_{2}, r_{3}\}} \Vert {\mathbf{X}} _{(1)} \Vert _{\ast }. \end{aligned}$$

Similarly, we have

$$\begin{aligned} \Vert \mathcal{X} \Vert _{\ast }\leq \sqrt{\min \{r_{3}, r_{1}\}} \Vert {\mathbf{X}} _{(2)} \Vert _{\ast }, \end{aligned}$$

and

$$\begin{aligned} \Vert \mathcal{X} \Vert _{\ast }\leq \sqrt{\min \{r_{1}, r_{2}\}} \Vert {\mathbf{X}} _{(3)} \Vert _{\ast }. \end{aligned}$$

Thus, the conclusion is obtained. □

### Factors affecting the upper bounds on the nuclear norm and further results

In this subsection, we discuss the factors affecting the nuclear norm of a tensor. Especially, we focus on the structure analysis of a tensor. Based on the discussion, some new upper bounds on the nuclear norms of tensors are presented.

Firstly, we give a simple example to illustrate that the nuclear norm of a tensor is closely related to the structure of this tensor.

#### Example 3.2

Let

18 $$A=\left[\begin{array}{cc} 0 & 1\\ 1 & 0 \end{array}\;|\;\begin{array}{cc} -1 & 0\\ 0 & 1 \end{array}\right].$$

Similar to the discussion of Example 3.1, since

$$\begin{aligned} \mathcal{A}=\mathbf{e}_{1;2}\circ \mathbf{e}_{2;2}\circ \mathbf{e} _{1;2}+\mathbf{e}_{2;2}\circ \mathbf{e}_{1;2} \circ \mathbf{e}_{1;2} +(- \mathbf{e}_{1;2})\circ \mathbf{e}_{1;2}\circ \mathbf{e}_{2;2}+ \mathbf{e}_{2;2} \circ \mathbf{e}_{2;2}\circ \mathbf{e}_{2;2} \end{aligned}$$

and

$$\begin{aligned} \langle \mathcal{A}, \mathbf{e}_{1;2}\circ \mathbf{e}_{2;2} \circ \mathbf{e}_{1;2}\rangle =&\langle \mathcal{A}, \mathbf{e}_{2;2}\circ \mathbf{e}_{1;2}\circ \mathbf{e}_{1;2}\rangle =\bigl\langle \mathcal{A}, (- \mathbf{e}_{1;2})\circ \mathbf{e}_{1;2}\circ \mathbf{e}_{2;2}\bigr\rangle \\ =&\langle \mathcal{A}, \mathbf{e}_{2;2} \circ \mathbf{e}_{2;2}\circ \mathbf{e}_{2;2}\rangle , \end{aligned}$$

then, according to the sufficient and necessary conditions of the nuclear norm decomposition obtained in [6], we get

$$\begin{aligned} \Vert \mathcal{A} \Vert _{\ast }=4. \end{aligned}$$

It is well known that the nuclear norm of a tensor is closely related to the number field [6]. Actually, the tensor $\mathcal{A}$ can be expressed as the following form:

$$A=\frac{1}{2}\left[\begin{array}{c} -1\\ -i \end{array}\right]\circ \left[\begin{array}{c} 1\\ i \end{array}\right]\circ \left[\begin{array}{c} i\\ 1 \end{array}\right]+\frac{1}{2}\left[\begin{array}{c} -1\\ i \end{array}\right]\circ \left[\begin{array}{c} 1\\ -i \end{array}\right]\circ \left[\begin{array}{c} -i\\ 1 \end{array}\right].$$

Let

$$W^{\left(1\right)}=\frac{1}{\sqrt{2}}\left[\begin{array}{cc} -1 & i\\ -1 & -i \end{array}\right],\;\;W^{\left(2\right)}=\frac{1}{\sqrt{2}}\left[\begin{array}{cc} 1 & -i\\ 1 & i \end{array}\right],\;\;W^{\left(3\right)}=\frac{1}{\sqrt{2}}\left[\begin{array}{cc} -i & 1\\ i & 1 \end{array}\right].$$

Then it holds

19 $$\left(W^{\left(1\right)},W^{\left(2\right)},W^{\left(3\right)}\right)\cdot A=\left[\begin{array}{cc} \sqrt{2} & 0\\ 0 & 0 \end{array}\;|\;\begin{array}{cc} 0 & 0\\ 0 & \sqrt{2} \end{array}\right].$$

For the sake of convenience, let $\hat{\mathcal{A}}=(\mathbf{W}^{(1)}, \mathbf{W}^{(2)}, \mathbf{W}^{(3)})\cdot \mathcal{A}$. Then, using the same method as above, we have

$$\begin{aligned} \langle \hat{\mathcal{A}}, \mathbf{e}_{1;2}\circ \mathbf{e}_{1;2} \circ \mathbf{e}_{1;2}\rangle =\langle \hat{\mathcal{A}}, \mathbf{e} _{2;2}\circ \mathbf{e}_{2;2}\circ \mathbf{e}_{2;2} \rangle . \end{aligned}$$

Thus $\Vert \hat{\mathcal{A}}\Vert _{\ast }=2\sqrt{2}$. Since all three matrices $\mathbf{W}^{(k)}$ ($1\leq k\leq 3$) are unitary matrices, based on the invariance of the Frobenius norm of a tensor under the multi-linear orthogonal transformations, we get

$$\begin{aligned} \Vert \mathcal{A} \Vert _{\ast }= \Vert \hat{\mathcal{A}} \Vert _{\ast }=2\sqrt{2}. \end{aligned}$$

**Figure 1:**
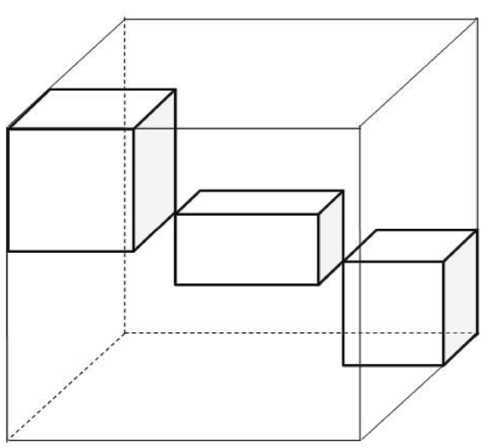
The block diagonal tensor with three diagonal blocks

Noting the structures of the tensors given by (18) and (19), the above derivation process implies that the nuclear norm of a tensor is closely related to the structure of this tensor. In what follows, we discuss the block diagonal tensor, which can be illustrated by Fig. 1. Furthermore, the block diagonal tensor can be expressed by using the direct sum operation “⊕” [4], which is defined as follows:

Let ${\mathcal{A}}=(a_{i_{1}\cdots i_{D}})\in \mathbb{R}^{n_{1}\times \cdots \times n_{D}}$ and ${\mathcal{B}}=(b_{j_{1}\cdots j_{D}}) \in \mathbb{R}^{n_{1}^{\prime}\times \cdots \times n_{D}^{\prime}}$, then the direct sum of ${\mathcal{A}}$ and ${\mathcal{B}}$ is an order-*D* tensor ${\mathcal{C}}=(c_{i_{1}\cdots i_{D}})={\mathcal{A}}\oplus {\mathcal{B}} \in \mathbb{R}^{(n_{1}+n_{1}^{\prime})\times \cdots \times (n_{D}+n_{D} ^{\prime})}$ defined by

$$\begin{aligned} c_{i_{1}\cdots i_{D}}= \textstyle\begin{cases} a_{i_{1}\cdots i_{D}}, &\text{if }1\leq i_{\alpha }\leq n_{\alpha }, \alpha =1,2,\ldots,D; \\ b_{i_{1}-n_{1},\ldots,i_{D}-n_{D}},&\text{if }n_{\alpha } +1\leq i _{\alpha }\leq n_{\alpha }+n_{\alpha }^{\prime}, \alpha =1,2,\ldots,D; \\ 0, &\text{otherwise}. \end{cases}\displaystyle \end{aligned}$$

Based on the discussion above, we present some properties of the spectral norm and nuclear norm of the tensor.

#### Lemma 3.1

*Let* $\mathcal{X}^{(l)}\in \mathbb{R}^{n_{1}^{(l)}\times \cdots \times n_{D}^{(l)}}$, $1\leq l\leq L$, *and*

$$\begin{aligned} \mathcal{X}=\mathcal{X}^{(1)}\oplus \cdots \oplus \mathcal{X}^{(L)} \in \mathbb{R}^{(\sum_{l=1}^{L}{n_{1}^{(l)}})\times \cdots \times ( \sum_{l=1}^{L}{n_{D}^{(l)}})}. \end{aligned}$$

*Then*

20 $$\begin{aligned} \Vert \mathcal{X} \Vert _{2}=\max _{1\leq l\leq L}\bigl\{ \bigl\Vert \mathcal{X}^{(l)} \bigr\Vert _{2}\bigr\} . \end{aligned}$$

#### Proof

According to the definition of the spectral norm of a tensor (Definition 2.3), it is easy to get

$$\begin{aligned} \Vert \mathcal{X} \Vert _{2}\geq \max_{1\leq l\leq L} \bigl\{ \bigl\Vert \mathcal{X}^{(l)} \bigr\Vert _{2}\bigr\} . \end{aligned}$$

Thus, the rest of the proof just needs to show

21 $$\begin{aligned} \Vert \mathcal{X} \Vert _{2}\leq \max _{1\leq l\leq L}\bigl\{ \bigl\Vert \mathcal{X}^{(l)} \bigr\Vert _{2}\bigr\} . \end{aligned}$$

Firstly, we consider the case of the third order tensors.

Suppose that $\mathcal{X}^{(l)}\in \mathbb{R}^{n_{1}^{(l)}\times n _{2}^{(l)}\times n_{3}^{(l)}}$ ($1\leq l\leq L$),

$$\begin{aligned} \mathcal{X}=\mathcal{X}^{(1)}\oplus \cdots \oplus \mathcal{X}^{(L)} \in \mathbb{R}^{(\sum_{l=1}^{L}{n_{1}^{(l)}}) \times (\sum_{l=1}^{L} {n_{2}^{(l)}})\times (\sum_{l=1}^{L}{n_{3}^{(l)}})}, \end{aligned}$$

and $\sigma \mathbf{u}\circ \mathbf{v}\circ \mathbf{w}$ is the best rank-one approximation of $\mathcal{X}$, where $\sigma =\Vert \mathcal{X} \Vert _{2}$, $\mathbf{u}\in \mathbb{R}^{\sum_{l=1}^{L}{n_{1}^{(l)}}}$, $\mathbf{v}\in \mathbb{R}^{\sum_{l=1}^{L}{n_{2}^{(l)}}}$, $\mathbf{w}=[ \mathbf{w}_{1}^{T},\ldots, \mathbf{w}_{L}^{T}]^{T}\in \mathbb{R} ^{\sum_{l=1}^{L}{n_{3}^{(l)}}}$, $\mathbf{w}_{l}\in \mathbb{R}^{n_{3} ^{(l)}}$ ($1\leq l\leq L$), and $\Vert \mathbf{u}\Vert _{2}=\Vert \mathbf{v}\Vert _{2}= \Vert \mathbf{w}\Vert _{2}=1$. Then the following matrix

$$X\times_{3}w^{T}=\left[\begin{array}{ccc} X^{\left(1\right)}\times_{3}w_{1}^{T} & & \\ & \ddots & \\ & & X^{\left(L\right)}\times_{3}w_{L}^{T} \end{array}\right]$$

is a block diagonal matrix. It follows

$$\begin{aligned} \Vert \mathcal{X} \Vert _{2}= \bigl\Vert \mathcal{X} \times_{3}\mathbf{w}^{T} \bigr\Vert _{2}= \max _{1\leq l\leq L}\bigl\{ \bigl\Vert \mathcal{X}^{(l)} \times_{3}\mathbf{w}_{l}^{T} \bigr\Vert _{2}\bigr\} \leq \max_{1\leq l\leq L}\bigl\{ \bigl\Vert \mathcal{X}^{(l)} \bigr\Vert _{2}\bigr\} . \end{aligned}$$

Hence, inequality (21) is proved. This also implies that equality (20) is true for the third order tensors.

Secondly, for the case of higher order tensors with order larger than or equal to four, the same result can be established by the recursive method.

In all, the conclusion is true. □

Then, based on Lemma 3.1, the following two results related to the nuclear norms of tensors can be established.

#### Lemma 3.2

*Let* $\mathcal{O}\in \mathbb{R}^{n_{1}^{(1)}\times \cdots \times n_{D} ^{(1)}}$ *be a zero tensor*, *and* $\mathcal{X}\in \mathbb{R}^{n_{1}^{(2)} \times \cdots \times n_{D}^{(2)}}$. *Then*

$$\begin{aligned} \Vert \mathcal{O}\oplus \mathcal{X} \Vert _{\ast }= \Vert \mathcal{X}\oplus \mathcal{O} \Vert _{\ast }= \Vert \mathcal{X} \Vert _{\ast }. \end{aligned}$$

#### Proof

Suppose that

$$\begin{aligned} \Vert \mathcal{X} \Vert _{\ast }=\sum_{p=1}^{P} \vert {\sigma_{p}} \vert \end{aligned}$$

and

$$\begin{aligned} \mathcal{X}=\sum_{p=1}^{P}{ \sigma_{p}{\mathbf{x}}_{p}^{(1)}\circ \cdots \circ {\mathbf{x}}_{p}^{(D)}}, \end{aligned}$$

where for all $1\leq p\leq P$, $\mathbf{x}_{p}^{(1)}\circ \cdots \circ {\mathbf{x}}_{p}^{(D)}$ are rank-one tensors with $\Vert {\mathbf{x}}_{p}^{(1)}\Vert _{2}=\cdots =\Vert {\mathbf{x}}_{p}^{(D)}\Vert _{2}=1$. Then

$$O⊕X=\sum_{p=1}^{P}\sigma_{p}\left(\begin{array}{c} x_{p}^{\left(1\right)}\\ 0 \end{array}\right)\circ \cdots \circ \left(\begin{array}{c} x_{p}^{\left(D\right)}\\ 0 \end{array}\right),$$

where all **0***s* denote zero vectors with suitable dimensions. This implies

$$\begin{aligned} \Vert \mathcal{O}\oplus \mathcal{X} \Vert _{\ast }\leq \Vert \mathcal{X} \Vert _{\ast }. \end{aligned}$$

Furthermore, assume that

$$\begin{aligned} \Vert \mathcal{X} \Vert _{\ast }=\langle \mathcal{X},\mathcal{Y} \rangle , \end{aligned}$$

where $\mathcal{Y}\in \mathbb{R}^{n_{1}^{(2)}\times \cdots \times n _{D}^{(2)}}$ and $\Vert \mathcal{Y}\Vert _{2}=1$.

Then, by Lemma 3.1, we have

$$\begin{aligned} \Vert \mathcal{O}\oplus \mathcal{Y} \Vert _{2}= \Vert \mathcal{Y} \Vert _{2}=1. \end{aligned}$$

It follows from Definition 2.5 that

$$\begin{aligned} \Vert \mathcal{O}\oplus \mathcal{X} \Vert _{\ast }\geq \langle \mathcal{O} \oplus \mathcal{X},\mathcal{O}\oplus \mathcal{Y}\rangle =\langle \mathcal{X},\mathcal{Y}\rangle = \Vert \mathcal{X} \Vert _{\ast }. \end{aligned}$$

Thus it holds $\Vert \mathcal{O}\oplus \mathcal{X}\Vert _{\ast }=\Vert \mathcal{X}\Vert _{\ast }$.

Using the same method, the equality $\Vert \mathcal{X}\oplus \mathcal{O} \Vert _{\ast }=\Vert \mathcal{X}\Vert _{\ast }$ can be proved. □

#### Lemma 3.3

*Let* $\mathcal{X}^{(l)}\in \mathbb{R}^{n_{1}^{(l)}\times \cdots \times n_{D}^{(l)}}$, $1\leq l\leq L$, *and*

$$\begin{aligned} \mathcal{X}=\mathcal{X}^{(1)}\oplus \cdots \oplus \mathcal{X}^{(L)} \in \mathbb{R}^{(\sum_{l=1}^{L}{n_{1}^{(l)}})\times \cdots \times ( \sum_{l=1}^{L}{n_{D}^{(l)}})}. \end{aligned}$$

*Then*

$$\begin{aligned} \Vert \mathcal{X} \Vert _{\ast }=\sum_{l=1}^{L}{ \bigl\Vert \mathcal{X}^{(l)} \bigr\Vert _{\ast }}. \end{aligned}$$

#### Proof

We just need to prove the case of $L=2$. For the general case, the conclusion can be obtained in a recursive way.

Let

$$\begin{aligned} \tilde{\mathcal{X}}_{1}=\mathcal{X}^{(1)}\oplus \mathcal{O}^{(1)}, \quad\quad \tilde{\mathcal{X}}_{2}= \mathcal{O}^{(2)}\oplus \mathcal{X}^{(1)}, \end{aligned}$$

where $\mathcal{O}^{(1)}\in \mathbb{R}^{n_{1}^{(2)}\times \cdots \times n_{D}^{(2)}}$ and $\mathcal{O}^{(2)}\in \mathbb{R}^{n_{1}^{(1)} \times \cdots \times n_{D}^{(1)}}$ are both zero tensors.

Then, by using Lemma 3.2, we get

22 $$\begin{aligned} \Vert \mathcal{X} \Vert _{\ast }= \Vert \tilde{ \mathcal{X}}_{1}+\tilde{\mathcal{X}} _{2} \Vert _{\ast }\leq \Vert \tilde{\mathcal{X}}_{1} \Vert _{\ast } + \Vert \tilde{\mathcal{X}}_{2} \Vert _{\ast }= \bigl\Vert \mathcal{X}^{(1)} \bigr\Vert _{\ast }+ \bigl\Vert \mathcal{X}^{(2)} \bigr\Vert _{\ast }. \end{aligned}$$

Suppose that

$$\begin{aligned} \bigl\Vert \mathcal{X}^{(l)} \bigr\Vert _{\ast }=\bigl\langle \mathcal{X}^{(l)},\mathcal{Y} ^{(l)}\bigr\rangle , \quad \text{and}\quad \mathcal{Y}^{(l)}\in \mathbb{R}^{n_{1} ^{(l)}\times \cdots \times n_{D}^{(l)}}, \bigl\Vert \mathcal{Y}^{(l)} \bigr\Vert _{2}=1. \end{aligned}$$

Then, by Lemma 3.1, we get

$$\begin{aligned} \bigl\Vert \mathcal{Y}^{(1)}\oplus \mathcal{Y}^{(2)} \bigr\Vert _{2}=1. \end{aligned}$$

Thus, according to Definition 2.5, we have

23 $$\begin{aligned} \begin{aligned}[b] \Vert \mathcal{X} \Vert _{\ast }&= \max_{ \substack{ \mathcal{Y}\in \mathbb{R}^{(n_{1}^{(1)}+n_{1}^{(2)})\times \cdots \times (n_{D}^{(1)}+n_{D}^{(2)})}\\ \Vert \mathcal{Y} \Vert _{2}=1 }}\bigl\{ \langle \mathcal{X},\mathcal{Y}\rangle \bigr\} \\ &\geq \bigl\langle \mathcal{X}^{(1)}\oplus \mathcal{X}^{(2)}, \mathcal{Y} ^{(1)}\oplus \mathcal{Y}^{(2)}\bigr\rangle \\ &= \bigl\Vert \mathcal{X}^{(1)} \bigr\Vert _{\ast }+ \bigl\Vert \mathcal{X}^{(2)} \bigr\Vert _{\ast }. \end{aligned} \end{aligned}$$

Combined (22) with (23), the results can be obtained. □

Based on the fact that the nuclear norm of a tensor is also kept invariant under the multi-linear orthogonal transformation, we get the following result.

#### Corollary 3.2

*Let* $\mathcal{X}\in \mathbb{R}^{n_{1}\times \cdots \times n_{D}}$. *If the tensor* $\mathcal{X}$ *admits a diagonal structure under the multi*-*linear orthogonal transformations*, *then*

$$\begin{aligned} \Vert \mathcal{X} \Vert _{\ast }=\sum_{p=1}^{P} \vert \sigma_{p} \vert , \end{aligned}$$

*where* $\sigma_{p}$ ($1\leq p\leq P$) *are the diagonal elements and* $P\leq n_{1}$.

This case presented by Corollary 3.2 is consistent with the definition of the nuclear norm of the matrix case, and in this case, the nuclear norm of the tensor can be accurately calculated.

Taking into account the structure information of the tensor, some new results of the upper bounds on the nuclear norms can be obtained. For the convenience of comparison, we just present the upper bounds on the nuclear norms of tensors through the dimensions of the tensors, without considering the orthogonal rank of the tensors.

#### Theorem 3.3

*Let* $\mathcal{X}\in \mathbb{R}^{n_{1}\times \cdots \times n_{D}}$ *and* *L* *be the maximum number of diagonal blocks that the tensor* $\mathcal{X}$ *can attain under the multi*-*linear orthogonal transformations*. *Suppose that the size of each diagonal block is* $n_{1}^{(l)}\times \cdots \times n_{D}^{(l)}$ *and*

$$\begin{aligned} \tilde{n}_{l}=\frac{\prod_{i=1}^{D}n_{i}^{(l)}}{\max \{n_{1}^{(l)} \cdots n_{D}^{(l)}\}},\quad 1\leq l\leq L. \end{aligned}$$

*Then it holds*

24 $$\begin{aligned} \Vert \mathcal{X} \Vert _{\ast }\leq \sqrt{ \sum_{l=1}^{L}\tilde{n}_{l}} \Vert \mathcal{X} \Vert _{F}. \end{aligned}$$

#### Proof

Assume that

$$\begin{aligned} \mathcal{X}=\mathcal{D}(\mathcal{X})\times_{1}{\mathbf{W}}^{(1)} \cdots \times_{D}{\mathbf{W}}^{(D)}, \end{aligned}$$

where

$$\begin{aligned} \mathcal{D}(\mathcal{X})=\mathcal{D}^{(1)}\oplus \cdots \oplus \mathcal{D}^{(L)} \end{aligned}$$

and $\mathbf{W}^{(d)}\in \mathbb{R}^{n_{d}\times n_{d}}$ ($1\leq d\leq D$) are orthogonal matrices, $\mathcal{D}^{(l)}\in \mathbb{R}^{n_{1}^{(l)} \times \cdots \times n_{D}^{(l)}}$, $1\leq l\leq L$.

Then it follows from the invariance of the Frobenius norm of a tensor under the multi-linear orthogonal transformation that

25 $$\begin{aligned} \Vert \mathcal{X} \Vert _{F}^{2}=\sum _{l=1}^{L} \bigl\Vert \mathcal{D}^{(l)} \bigr\Vert _{F}^{2}. \end{aligned}$$

Furthermore, since the nuclear norm of a tensor is also kept invariant under the multi-linear orthogonal transformation, we get

$$\begin{aligned} \Vert \mathcal{X} \Vert _{\ast }= \bigl\Vert \mathcal{D}( \mathcal{X}) \bigr\Vert _{\ast }. \end{aligned}$$

Hence, by Lemma 3.3 and (25), we get

$$\begin{aligned} \begin{aligned} \Vert \mathcal{X} \Vert _{\ast }&= \bigl\Vert \mathcal{D}(\mathcal{X}) \bigr\Vert _{\ast } \\ &=\sum_{l=1}^{L}{ \bigl\Vert \mathcal{D}^{(l)} \bigr\Vert _{\ast }} \\ &\leq \sum_{l=1}^{L}{\sqrt{ \tilde{n}_{l}} \bigl\Vert \mathcal{D}^{(l)} \bigr\Vert _{F}} \\ &\leq \sqrt{\sum_{l=1}^{L} \tilde{n}_{l}}\sqrt{\sum_{l=1}^{L}{ \bigl\Vert \mathcal{D}^{(l)} \bigr\Vert _{F}^{2}}} \quad (\text{by Cauchy-Schwarz inequality}) \\ &=\sqrt{\sum_{l=1}^{L} \tilde{n}_{l}} \Vert \mathcal{X} \Vert _{F}. \end{aligned} \end{aligned}$$

 □

Without loss of generality, suppose that

$$\begin{aligned} n_{D}=\max \{n_{1},\ldots, n_{D}\}. \end{aligned}$$

Since

$$\begin{aligned}& n_{1}=\sum_{l=1}^{L}n_{1}^{(l)} \\& \vdots \\& n_{D-1}=\sum_{l=1}^{L}n_{D-1}^{(l)}, \end{aligned}$$

it is easy to get

$$\begin{aligned} \begin{aligned} \frac{\prod_{i=1}^{D}n_{i}}{\max \{n_{1},\ldots, n_{D}\}}&=\prod_{i=1} ^{D-1}n_{i} \\ &=\Biggl(\sum_{l=1}^{L}n_{1}^{(l)} \Biggr)\cdots \Biggl(\sum_{l=1}^{L}n_{D-1}^{(l)} \Biggr) \\ &\geq \sum_{l=1}^{L}\tilde{n}_{l}. \end{aligned} \end{aligned}$$

Thus, the upper bound given by (24) improves (1). Theorem 3.3 also shows that the upper bound on the nuclear norm can be improved by using the structural information.

Similarly, the following upper bound can also be obtained.

#### Theorem 3.4

*Let* $\mathcal{X}\in \mathbb{R}^{n_{1}\times \cdots \times n_{D}}$, *and* *L* *be the maximum number of diagonal blocks that the tensor* $\mathcal{X}$ *can attain under the multi*-*linear orthogonal transformations*. *Suppose that the size of each diagonal block is* $n_{1}^{(l)}\times \cdots \times n_{D}^{(l)}$, *and*

$$\begin{aligned} \tilde{n}_{l}=\frac{\prod_{i=2}^{D}n_{i}^{(l)}}{\max \{n_{2}^{(l)} \cdots n_{D}^{(l)}\}},\quad 1\leq l\leq L, \end{aligned}$$

*and*

$$\begin{aligned} \tilde{n}=\max_{1\leq l\leq L}\{\tilde{n}_{l}\}. \end{aligned}$$

*Then it holds*

26 $$\begin{aligned} \Vert \mathcal{X} \Vert _{\ast }\leq \sqrt{\tilde{n}} \Vert \mathbf{X}_{(1)} \Vert _{\ast }. \end{aligned}$$

#### Proof

Similar to the proof of Theorem 3.3, assume

$$\begin{aligned} \mathcal{X}=\mathcal{D}(\mathcal{X})\times_{1}{\mathbf{W}}^{(1)} \cdots \times_{D}{\mathbf{W}}^{(D)}, \end{aligned}$$

where

$$\begin{aligned} \mathcal{D}(\mathcal{X})=\mathcal{D}^{(1)}\oplus \cdots \oplus \mathcal{D}^{(L)} \end{aligned}$$

and $\mathbf{W}^{(d)}\in \mathbb{R}^{n_{d}\times n_{d}}$ ($1\leq d\leq D$) are orthogonal matrices.

Then it holds

$$\begin{aligned} \begin{aligned} \Vert \mathcal{X} \Vert _{\ast }&= \bigl\Vert \mathcal{D}(\mathcal{X}) \bigr\Vert _{\ast } \\ &=\sum_{l=1}^{L} \bigl\Vert \mathcal{D}^{(l)} \bigr\Vert _{\ast } \\ &\leq \sum_{l=1}^{L}\sqrt{ \tilde{n}_{l}} \bigl\Vert \mathbf{D}^{(l)}_{(1)} \bigr\Vert _{\ast } \\ &\leq \sqrt{\tilde{n}} \sum_{l=1}^{L} \bigl\Vert \mathbf{D}_{(1)}^{(l)} \bigr\Vert _{ \ast } \\ &\leq \sqrt{\tilde{n}} \sum_{l=1}^{L} \Vert \mathbf{D}_{(1)} \Vert _{\ast } \\ &=\sqrt{\tilde{n}} \Vert \mathbf{X}_{(1)} \Vert _{\ast }. \end{aligned} \end{aligned}$$

 □

Theorem 3.4 implies that the upper bound given by nuclear norms of the unfolding matrices is more closely related to the structure. For the sake of clarity, we give a simple example to illustrate.

#### Example 3.3

Let $\mathcal{A}$ be defined in Example 3.2, and

$$\begin{array}{rl} B & =A⊕A\\ & =[\begin{array}{cccc} 0 & 1 & 0 & 0\\ 1 & 0 & 0 & 0\\ 0 & 0 & 0 & 0\\ 0 & 0 & 0 & 0 \end{array}\;\left|\;\begin{array}{cccc} -1 & 0 & 0 & 0\\ 0 & 1 & 0 & 0\\ 0 & 0 & 0 & 0\\ 0 & 0 & 0 & 0 \end{array}\;\right|\;\begin{array}{cccc} 0 & 0 & 0 & 0\\ 0 & 0 & 0 & 0\\ 0 & 0 & 0 & 1\\ 0 & 0 & 1 & 0 \end{array}\;\left|\;\begin{array}{cccc} 0 & 0 & 0 & 0\\ 0 & 0 & 0 & 0\\ 0 & 0 & -1 & 0\\ 0 & 0 & 0 & 1 \end{array}\right]. \end{array}$$

Then, by Theorem 1.2, we get

$$\begin{aligned} \Vert \mathcal{B} \Vert _{\ast }\leq \sqrt{4*4} \Vert \mathcal{B} \Vert _{F}=8 \sqrt{2}. \end{aligned}$$

It follows from Theorem 3.4 that

$$\begin{aligned} \Vert \mathcal{B} \Vert _{\ast }\leq \sqrt{2*2} \Vert \mathcal{B} \Vert _{F}=4 \sqrt{2}. \end{aligned}$$

There has been a marked improvement in the upper bounds on the nuclear norm.

## Conclusions

In this paper, we provide a new estimation method for the upper bounds on the nuclear norms and obtain some new upper bounds related to the nuclear norms. Meanwhile, it is found that the upper bounds on the nuclear norms are not only related to the dimensions of the tensor but also to the structure of the tensor. Taking into consideration the structure information of the tensor, the upper bounds on the nuclear norms can be improved.
